# Regulation of whole-transcriptome sequencing expression in COPD after personalized precise exercise training: a pilot study

**DOI:** 10.1186/s12931-023-02461-y

**Published:** 2023-06-13

**Authors:** Panpan Liu, Meilan Zhang, Hongchang Gao, Shaojun Han, Jinming Liu, Xingguo Sun, Lei Zhao

**Affiliations:** 1 Department of Pulmonary and Critical Care Medicine, Shanghai Pudong New Area Gongli Hospital, 219 MiaoPu Road, Shanghai, 200315 People’s Republic of China; 2 Department of Physiology and Medicine, Fuwai Hospital, Chinese Academy of Medical Sciences National Center of Cardiovascular Diseases, Beijing, People’s Republic of China; 3 Department of Pulmonary and Critical Care Medicine, Shanghai Pulmonary Hospital Affiliated to TongJi University, Shanghai, China

**Keywords:** Whole-transcriptome sequencing, Aerobic training, COPD, CeRNA network

## Abstract

**Background:**

Chronic obstructive pulmonary disease (COPD) is one of the world’s leading causes of death and a major chronic respiratory disease. Aerobic exercise, the cornerstone of pulmonary rehabilitation, improves prognosis of COPD patients; however, few studies have comprehensively examined the changes in RNA transcript levels and the crosstalk between various transcripts in this context. This study identified the expression of RNA transcripts in COPD patients who engaged in aerobic exercise training for 12 weeks, and further constructions of the possible RNAs networks were made.

**Methods:**

Peripheral blood samples for all four COPD patients who benefited from 12 weeks of PR were collected pre- and post-aerobic exercises and evaluated for the expression of mRNA, miRNA, lncRNA, and circRNA with high-throughput RNA sequencing followed by GEO date validation. In addition, enrichment analyses were conducted on different expressed mRNAs. LncRNA-mRNA and circRNA-mRNA coexpression networks, as well as lncRNA-miRNA-mRNA and circRNA-miRNA-mRNA competing expression networks (ceRNAs) in COPD were constructed.

**Results:**

We identified and analyzed the differentially expressed mRNAs and noncoding RNAs in the peripheral blood of COPD patients’ post-exercise. Eighty-six mRNAs, 570 lncRNAs, 8 miRNAs, and 2087 circRNAs were differentially expressed. Direct function enrichment analysis and Gene Set Variation Analysis showed that differentially expressed RNAs(DE-RNAs) correlated with several critical biological processes such as chemotaxis, DNA replication, anti-infection humoral response, oxidative phosphorylation, and immunometabolism, which might affect the progression of COPD. Some DE-RNAs were validated by Geo databases and RT-PCR, and the results were highly correlated with RNA sequencing. We constructed ceRNA networks of DE-RNAs in COPD.

**Conclusions:**

The systematic understanding of the impact of aerobic exercise on COPD was achieved using transcriptomic profiling. This research offers a number of potential candidates for clarifying the regulatory mechanisms that exercise has on COPD, which could ultimately help in understanding the pathophysiology of COPD.

**Supplementary Information:**

The online version contains supplementary material available at 10.1186/s12931-023-02461-y.

## Introduction

Chronic obstructive pulmonary disease (COPD) is one of the top three causes of death worldwide [1]. COPD is characterized by persistent respiratory symptoms and airflow limitations that are due to airway or alveolar abnormalities [1]. High morbidity and mortality rates have affected more than 700 billion people, including nearly 100 million people in China [2]. As the global population ages, morbidity and mortality rates are expected to increase. In the past few decades, pharmacological therapies for COPD have improved; examples include antibiotics, triple inhaled therapy, and alpaha-1 antitrypsin augmentation therapy. However, prognosis for COPD patients remains challenging due to high levels of heterogeneity of disease [3]. Further complementary therapies are essential to improve the clinical outcomes of COPD patients.

Exercise is used to maintain and restore homeostasis at organismal, tissue, cellular, and molecular levels. It has the potential to prevent or inhibit a wide range of illnesses, including COPD [4]. Exercise immunology research has revealed that both acute and ongoing exercise have a significant impact on the immune system, especially immune metabolism [5]. Regular exercise mediates an anti-inflammatory and antioxidant state [6, 7] and the benefits to COPD patients are considerable [8]. Aerobic exercise is a cornerstone of pulmonary rehabilitation(PR) to improve health-related quality of life and exercise capacity, as well as reduce dyspnea, hospitalization, exacerbation, and mortality [9–11]. Some studies suggest that exercises can reduce chronic inflammation, improve the diaphragm and cognitive function, and reverse airway remodeling [12–15]. However, despite exercise’s profound benefits for treating COPD, knowledge of how exercise improves health and the molecular mechanism of immunometabolism response to exercise remains limited [16, 17]. Furthermore, physiological responses to exercise vary between individuals because of the heterogeneous phenotype of COPD, exercise modalities, and levels of intensity [18]. Additional studies on the molecular mechanisms of exercise intervention in COPD, coupled with advances in the characterization of the human genome, may improve personalized exercise interventions and offer new insight into treatment strategies.

Previous studies have shown molecular insights into the advantages of exercise for people with COPD. For example, nuclear receptor subfamily 4 group A member 3 (NR4A3) induced metabolic responses in skeletal muscle post-exercises [19], and chemerin improved the diaphragm function by regulating inflammation and metabolism of COPD^5^. Other metabolic diseases and secondary ageing [20] were also precluded and ameliorated by exercise, and wide-scale use of.multi-omics approaches helped illuminate genomic regulation in response to exercise. Most public data referenced skeletal muscle transcriptomics and relevant phosphorylation cascades that activated metabolic enzymes such as AKT and AMPK [21], as well as alterations in DNA structures [22]. Further, although there are few predictive tools to access the exercise response for patients, MetaMEx (https://metamex.eu/) provides the most extensive dataset of skeletal muscle transcription and an online interface to readily interrogate the database [19]. Nevertheless, muscle samples were difficult to obtain from patients. Plasma samples are more convenient and more readily accepted by patients; however, no study has systematically evaluated the alteration of RNA expression in peripheral blood leucocyte in response to personalized exercise for COPD treatment. Therefore, we aimed to conduct a systems-level analysis of the therapeutic mechanism of personalized precise exercise training (PPET).

High-throughput molecular biological techniques, including a transcriptomics approach, have been used to explore complex biological processes and the role of exercise in systems biology. We used whole-transcriptome sequencing to explore responses to PPET [23], which were more accepted by patients with exertional dyspnea. Subsequently, a differential expression analysis of mRNA, miRNA, lncRNA, and circRNA (DE-mRNAs, DE-miRNAs, DE-lncRNAs and DE-circRNAs) was performed between pre-and post-exercise groups followed by functional enrichment and interaction prediction analysis. In addition, the results were validated using GEO data. This study may shed light on a novel exercise program that is suitable for a number of COPD patients and may also identify potential biomarkers with various prognostic and therapeutic implications.

## Methods

### Patients and exercise training protocol

Four COPD patients who benefited from 12 weeks of PR were recruited from clinical cohort research (registration number: ChiCTR2100053232) in Pudong New Area Gongli Hospital. COPD was diagnosed according to the Global Initiative for Chronic Obstructive Lung Disease (GOLD) criteria. The GOLD pulmonary function criterion for COPD diagnosis was post-bronchodilator forced expiratory volume in 1 s (FEV1)/ forced vital capacity (FVC) ratio < 0.7. All subjects had no significant cardiac dysfunction, active infection (e.g., hepatitis, tuberculosis), or exercise contraindications such as neurological or psychiatric disorders. The study was approved by the ethics committee of Shanghai Pudong New Area Gongli Hospital and all participants were provided with written informed consent for use of their blood samples for scientific purposes.

Patients were trained on Cycle Ergometer (Qianjing 20,003, China) for three days with different adaptive loads, based on the results of the cardiopulmonary exercise test (CPET) and continuous functional tests. Exercise intensity was individualized moderate intensity, and objectively and quantitatively formulated with CPET (Δ50% load ± 10 Watt) [24], with Δ50% load = (load at anaerobic threshold – increasing load per minute × 0.75 / 2 + (peak load – increasing load per minute × 0.75) / 2. After the adaptive process, exercise began on the fourth day and lasted for 12 weeks. Exercise frequency was determined according to the individualized response: 1 ~ 4 times / day, 5 ~ 7 days / week. Patients warmed up at a load of zero watts for five minutes and then at a personalized load sustained for 30 min of effective exercise (if revolutions per minute (RPM) < 60, patients could rest then continue the exercise).

### Sample preparation

Three milliliters of fresh whole blood were harvested from pre- and post-exercise COPD patients. Peripheral blood leucocytes were isolated from 3 ml of fresh whole blood within two hours of collection, by Pancoll gradient centrifugation of one collected Vacutainer EDTA-tube, then frozen in liquid nitrogen and stored at – 80 °C for further studies.

### RNA isolation and library preparation

Total RNA was extracted using the TRIzol reagent according to the manufacturer’s protocol. RNA purity and quantification were evaluated using the NanoDrop 2000 spectrophotometer (Thermo Fisher Scientific, Waltham, MA, USA). RNA integrity was assessed using the Agilent 2100 Bioanalyzer (Agilent Technologies, Santa Clara, CA, USA). The libraries were constructed using TruSeq Stranded Total RNA with Ribo-Zero Gold (illumina, Cat.No. RS-122-2301) according to the manufacturer’s instructions.

### RNA sequencing and differentially expressed RNAs analysis

The libraries were sequenced on an Illumina HiSeq X Ten platform, and 150 bp paired-end reads were generated. Approximately 95 million raw reads for each sample were generated. Raw data (raw reads) of fastq format were first processed using the Trimmomatic software [25]. In this step, clean data (clean reads) were obtained by removing reads containing adapter and ploy-N or low quality reads from raw data. Approximately 93 million clean reads for each sample were retained for subsequent analyses.

Sequencing reads were mapped to the human genome (GRCh38) using HISAT2 [26]. For mRNAs, FPKM [27] of each gene was calculated using Cufflinks [28], and the read counts of each gene were obtained by HTSeq-count [29]. Differential expression analysis was performed using the DESeq (2012) R package [30]. P-value < 0.05 was set as the threshold for significant differential expression. For lncRNAs, the transcriptome from each dataset was assembled independently using the Cufflinks 2.0 program [28]. All transcriptomes were pooled and merged to generate a final transcriptome using Cuffmerge (Cufflinks 2.0). All transcripts that overlapped with known mRNAs, other non-coding RNA, and non-lncRNA were discarded. Next, the transcripts longer than 200 bp and the number of exons > 2 were selected, and the CPC (v 0.9-r2) [31], PLEK (v 1.2) [32], CNCI (v 1.0) [33], Pfam (v 30) [34] were used to predict transcripts with coding potential. The novel predicted lncRNAs were obtained through these processes. The characteristics (including length, type, number of exons) of lncRNA were analyzed after screening. Then, the novel predicted lncRNAs and known lncRNAs (from NCBI and Ensemble database) were used for expression calculations and differential screening. circRNAs were identified using CIRI (v2.0.3) [35] and the expression of circRNAs were calculated using RPM (spliced reads per millon mapping) [9]. Differential expression analysis was completed using the DESeq (2012) R package. All sequencing processes and analyses were performed by OE Biotech Co., Ltd. (Shanghai, China).

### Gene Ontology (GO) term and KEGG pathway analysis

The gene list of DE-mRNAs were uploaded to the Database for Annotation, Visualization, and Integrated Discovery (DAVID, https://david.ncifcrf.gov/), which is a comprehensive set of functional annotation tools for researchers to understand biological meaning behind large sets of genes The official gene symbol was selected as an identifier, and homo sapiens was selected as the species. Finally, Gene Ontology (GO) analysis and Kyoto Encyclopedia of Genes and Genomes (KEGG) pathway analysis enrichment results were obtained [36, 37]. With the enriched gene count ≥ 2 and p < 0.05 significance threshold, GO terms and pathways were considered significant. The top five results in ascending order were displayed in this study.

### Gene set variation analysis (GSVA)

The gene list for each biological function was obtained from the AmiGO2 portal (http://amigo.geneontology.org). The biological functional enrichment score of each patient was calculated by Gene Set Variation Analysis (GSVA) analysis, using GSVA package (R environment) under default parameters [38]. Diverging bars of the enrichment results was drawn with the package (R environment).

### PPI network and module analysis

The interaction between DE-mRNA encoded proteins was analyzed by STRING (version 10.0, https://string-db.org/cgi/input.pl) database. We input all DE-mRNA sets, and the species was set as human. The PPI network was built by Cytoscape software (version 3.9.1; https://cytoscape.org/). The Cytoscape’s plug-in MCODE [39] was used to examine the PPI network's most important clustering modules (version2.0.0). We set the PPI score parameter to 0.7 to obtain the interaction pairs that were most closely related. The threshold for the significant clustering module gene was score ≥ 2. GO enrichment analysis was conducted for the top 10 clustering module genes. The GO terms with enriched gene count ≥ 2 and the significance threshold p < 0.05 were considered significant.

### Co-expression mRNAs of DE-cirRNA and DE-LncRNA

The Pearson's correlation coefficients of each DE-mRNA and DE-lncRNA and each DE-mRNA and DE-circRNA were calculated. The cor function in R software was used to calculate these correlation coefficients. A screen of |R|> 0.9 and p < 0.05 was used for co-expression relationships.

### ceRNA network construction

Competing endogenous RNAs(ceRNAs) are the lncRNAs, circRNAs, and mRNAs that competitively bind miRNAs and act as miRNA sponges. The lncRNA, mRNAs, and circRNA regulatory relationships with DE-miRNA were predicted using the StarBase (http://starbase.sysu.edu.cn/). The lncRNA, circRNAs and mRNA that were substantially differently expressed and regulated by the same miRNA were screened, using DE-lncRNAs, miRNAs, and mRNAs as well as regulatory relationships of DE-miRNA that were predicted using the StarBase. The lncRNA-miRNA-mRNA and circRNA-miRNA-mRNA networks were constructed with Cystoscope software v3.8.0 (San Diego, CA, USA) to investigate the role and interactions between ncRNAs and mRNAs after rehabilitation treatment.

### Data validation

GEO data were used for RNA data validation (GSE76705 for mRNAs validation, GSE24709 and GSE 61741are for miRNAs validation). MiRNAs and mRNAs expression matrices and annotation information were downloaded from GEO database separately. The matrices were submitted to a differential expression analysis in COPDs against normal controls using the limma R package [40]. A criteria for substantial DE-mRNA and DE-miRNA was defined as p-value 0.05. Expression levels of each genes were performed using GraphPad 8.

### RT-PCR for DE-RNAs

cDNA was synthesized using a TransScript All-in-One First-Strand cDNA Synthesis SuperMix (Transgen Biotech, Beijing, China), was performed. PCR was performed using a Bio-Rad PCR instrument (Bio-Rad, Hercules, CA, U.S.A.) with 2 × Taq PCR Master Mix (Solarbio, Beijing, China) following the manufacturer’s instructions. The fold changes were calculated by means of relative quantification (2 − △△Ct method). PCR primers are described as below: mirRNA 144: forward 5′-UUCAAUCAACUUUACUGUAA-3′and reverse 5′-UCAUGUAGUAUAUGACAU-3′; CCL23: forward 5′-CATCTCCTACACCCCACGAAG-3′and reverse 5′-GGGTTGGCACAGAAACGTC-3′; CPA3: forward 5′-GGGTTTGATTGCTACCACTCTT-3′and reverse 5′-GCCAAGTCCTTTATGATGTCTGC-3′; PLCB4: forward 5′- TTGACAGATACGAGGAGGAATCC-3′and reverse 5′GAGGGAGCATTCTAGCACCTG-3′; IGF2R: forward 5′- GCTTTGACAGCGAGAATCCC-3′and reverse 5′-TCCTACAGCAAGTGGTCAGC-3′.

### Statistical analysis

R 4.1.3 was used for bioinformatics analysis. Data processing and analysis were performed using GraphPad 8 (GraphPad Software, Inc., La Jolla, CA, USA, www. graphpad.com).We used paired Student t-test for clinical characters to analyze the differences between groups with double tail test. P < 0.05 was considered statistically significant.

## Results

### Individualized aerobic exercise training improved cardiopulmonary function of COPD patients

Patients performed a gradually increasing work rate CPET to maximal tolerance on an electromagnetically braked cycle ergometer in the upright position. Individualized training load was decided according to the CPET results. Aerobic exercise training lasted for 12 weeks, and peripheral blood samples were harvested pre- and post-12-week exercise training. Clinical characters of included patients are presented in (Table 1). The peak VO_2_, peak VO_2_/kg, VO_2_ at AT, peak workload, and six meter walk time (6MWT) were evaluated to assess the exercise capacity. The oxygen pulse, resting and peak HR, and nocturnal oxygen saturation were used to assess the cardiac function. CAT scores and pulmonary function tests were used for assessing respiratory symptoms.

**Table 1:** Clinical information of patients

	**sample-1**	**sample-2**	**sample-3**	**sample-4**
*Demographic data*				
Age, years	65.00	67.00	64.00	60.00
BMI, Kg/m^2^	16.22	28.28	27.13	14.69
Gender	Male	Male	Male	Male
*Smoking history*				
Current smoker	N	N	N	N
Ex-smoker	Y	N	Y	Y
Smoking, (pack-years)	7.5	0.00	15	20
*Lung function*				
FEV1, %pred	64.8	36.7	31.9	/
FEV1/FVC, %	65.33	55.14	41.3	/
RV, %pred	78.5	123	112	/
TLC, %pred	79.7%	82.1	75.3	/
RV/TLC, %	37.6	59.85	52.45	/
DLCO, %pred	65	65.5	49	/
CAT score				
	32	22	24	35
*Complication*				
	Osteoporosis	Asthma Hypertension	Hypertension	Type II RF Hyperuricemia

*BMI* body mass index, *FEV1* forced expiratory volume in one second, *RV* residual volume, *TLC* total lung capacity, *DLCO* carbon monoxide diffusion capacity, *CAT* COPD assessment test. ”/”, patients failed to complete the measurements of pulmonary function test

**Fig. 1:**
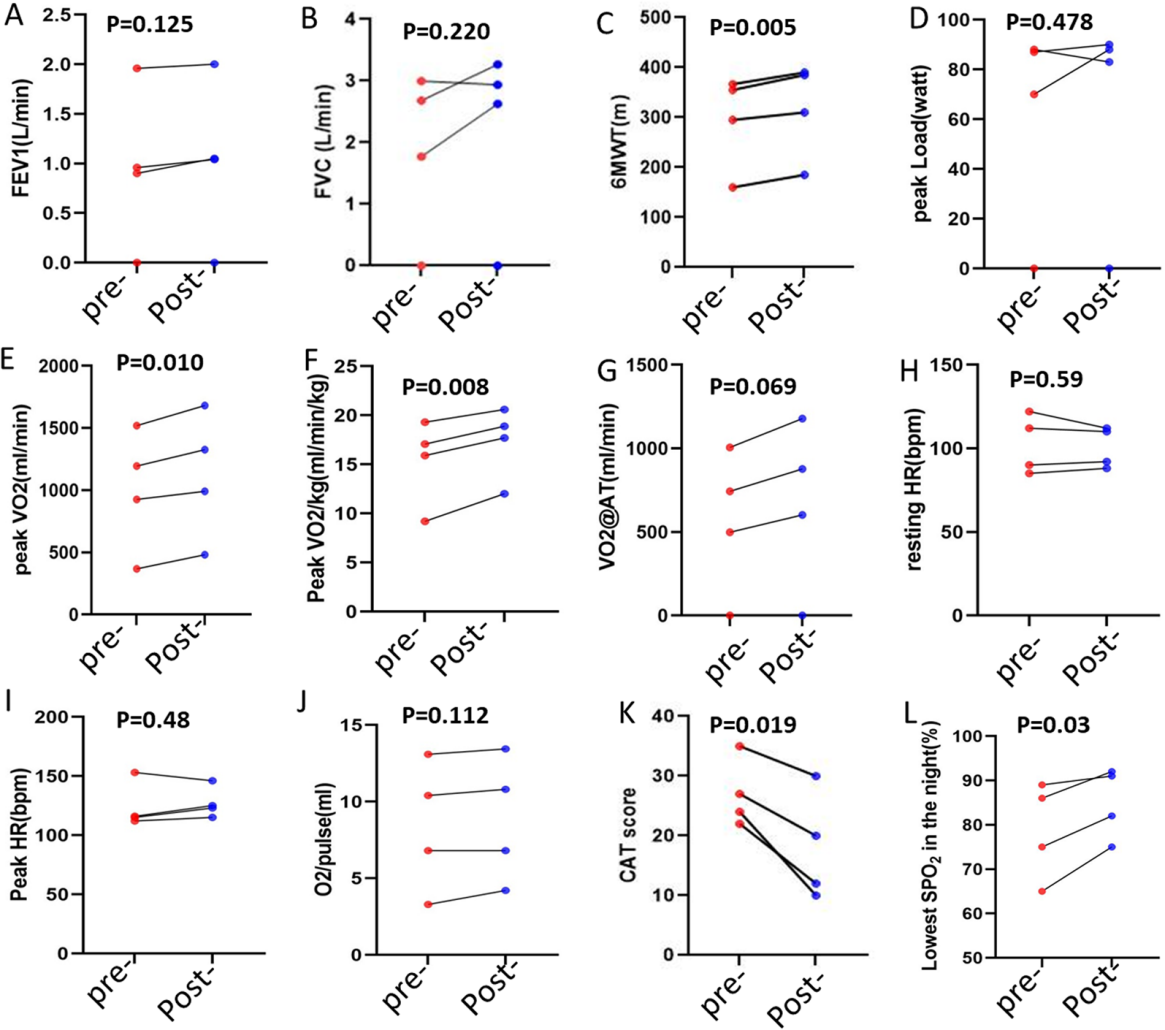
Changes in clinical parameters of patients pre- and post-exercise. 6 min walking distance (**C**), oxygen consumption at peak exercise (**E**), peak VO2/pulse (**F**); CAT score of common symptoms: CAT score (**K**) and nocturnal oxygen saturation (**L**) are significantly changed; FVC (**A** & **B**), oxygen uptake anaerobic threshold (**G**), working load at peak exercise (**D**), cardiac function by CPET: oxygen pulse (**J**), resting and peak heart rate (**H** & **I**) are not changed significantly. *FEV1* forced expiratory volume during the first second, *FVC* forced vital capacity, *VO2* oxygen consumption, *HR* heart rate, *SPO2* oxygen saturation, *@AT* at anaerobic threshold, “pre-” & “post” means pre- or post-12 weeks exercises in hospital

Regarding exercise tolerance, there was a significant increase in the Peak VO2 or Peak VO2/kg, after exercise training (102 ml/min, 95% CI: 57.12–146.90, t = 7.23, P = 0.005; 1.78 ml/min/kg, 95% CI: 0.61–2.94, t = 4.85, P = 0.017; respectively), with an improved tread of VO2 at AT or VO2/Kg at AT (89.50 ml/min, 95% CI: − 7.43–186.40, t = 2.94, P = 0.07; 1.35 ml/min/kg, 95% CI: − 0.18–2.87, t = 2.82, P = 0.07). After exercise training, the 6MWT had significantly improved (23.25 m, 95% CI: 13.32–33.18, t = 7.454, P = 0.005). However, the peak workloads during exercise did not change significantly (1.5-Watt, 95% CI: -7.17–10.17, t = 0.56, P = 0.62). After 12 weeks of exercise training, cardiac functions such as the oxygen pulse, resting and peak HR were unaffected. (1.55, 95% CI: 0.40–2.70, P = 0.008; − 1.75 bpm, 95% CI: -11.15–7.66, t = 0.59, P = 0.59; 2.75 bpm, 95% CI:-8.149–13.64, t = 0.80, P = 0.48). As a result of exercise training, the nocturnal oxygen saturation demonstrated an increase trend (5.75%, 95% CI: − 0.92–12.42, t = 2.74, P = 0.07) (Fig. 1).

Significant improvements in CAT scores were observed in all patients (− 9, 95% CI: − 15.23–2.77, t = 4.60, P = 0.019), but pulmonary functions including FEV1 and FVC did not change, while two observers’ FEV1 and FVC improved. As expected, the results suggested that individualized aerobic training increased exercise tolerance and improved respiratory symptoms.

### Differential expression analysis

**Fig. 2:**
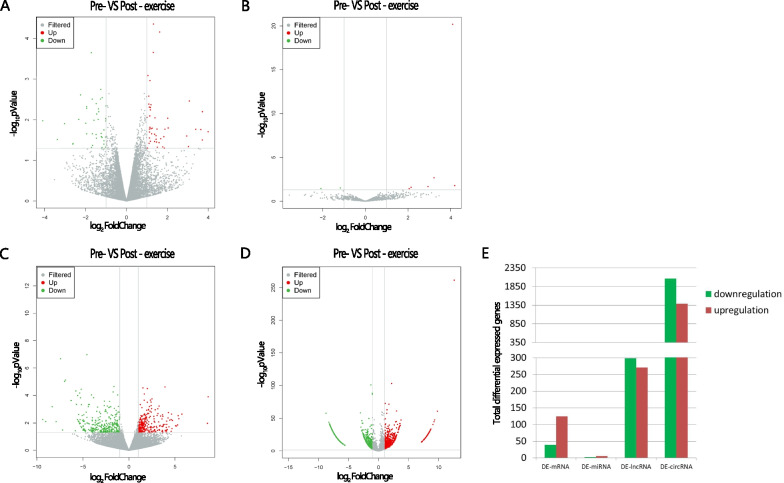
Volcanomap of differential expressed molecules. Comparison of differential expressed differtially expressed mRNAs (**A**), differentially expressed miRNAs (**B**), differentially expressed lncRNAs (**C**), and differentially expressed circRNAs (**D**) in pre- and post-exercise COPD groups. Total numbers of DE-molecules were shown in (**E**). Red dots represent upregulation, green represent downregulattion and gray represent non-differential. Differentiallly expressed molecules were identified as P < 0.05 and log2 [fold change] > 1

We used high-throughput RNA sequencing technology to examine the transcriptome of COPD patients' peripheral blood leukocytes pre-and post-exercise training in search of relevant biomarkers and important mechanisms. According to the screening criteria (log_2_ FoldChange ≥ 1.0 and p-values < 0.05), 86 DE-mRNAs were obtained, forty-seven were up-regulated and 39 were down-regulated. Six of the eight DE-miRNAs were up-regulated, and two were down-regulated. 570 DE-lncRNAs in total were found, of which 271 were up-regulated and 299 were down-regulated. 2064 DE-circRNAs in total were discovered, of which 676 showed up-regulation and 1388 showed down-regulation. The violin graphic of DE-mRNA, DE-miRNA, DE-lncRNA, and DE-circRNA is illustrated in Fig. 2. Our study offered a thorough explanation of how routine exercise affects the entire transcriptome in COPD patients.

### Functional enrichment analysis of DE-mRNAs

**Fig. 3:**
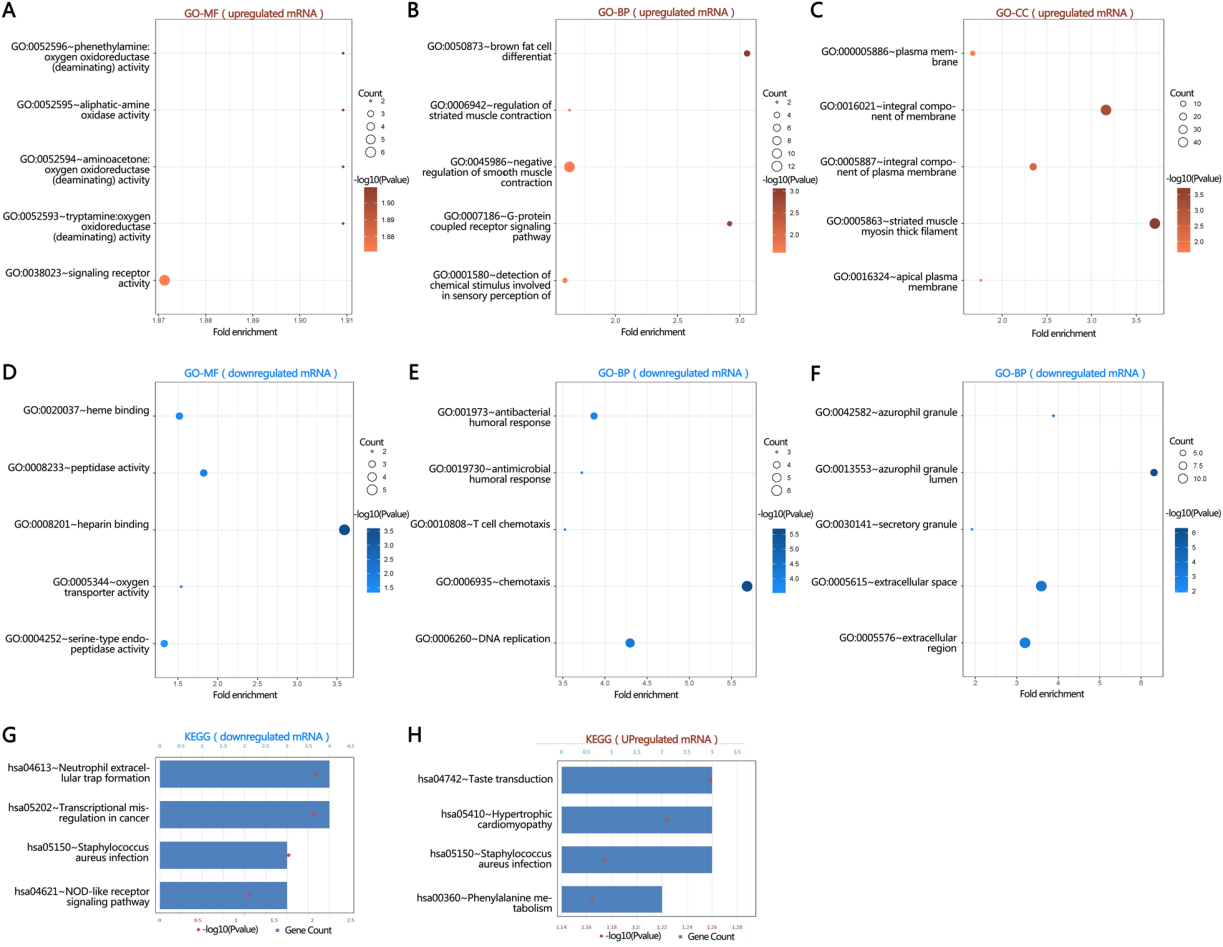
Enrichment analysis of DE-mRNAs. **A**–**F** Top 5 molecular unctions (MF), biological process (BP) terms and cellular components (CC) enriched by unregulated and down regulated genes (DE-mRNAs) separately. **G**, **H** The enriched Kyoto Encyclopedia of Genes and Genomes (KEGG) pathway by DE-mRNAs

We performed Gene Ontology and KEGG analysis on the 86 DE-mRNAs (screening standards: log_2_ FoldChange ≥ 1 and p-values < 0.05). Related biological processes (BP), cellular components (CC), molecular functions (MF), and signaling pathways are revealed in Fig. 3. Up-regulation genes are enriched with 14 terms from the GO-BP, five terms from CC, 15 terms from MF (Fig. 3A–C); while down-regulation genes are enriched with 30 terms from GO-BP, nine terms from CC, and nine terms from MF (Fig. 3D–F). From the BP analysis results, we found that the up-regulated genes were mainly involved in brown fat cell differentiation, regulation of striated muscle contraction, negative regulation of smooth muscle contraction, G-protein coupled receptor signaling pathways, and detection of chemical stimuli involved in sensory perception of bitter taste. Additionally, the down-regulated genes were significantly associated with chemotaxis, DNA replication, antibacterial humoral response, antimicrobial humoral response, and T-cell chemotaxis. CC analysis showed that DE-mRNA target genes were enriched in plasma membrane, zurophil granule lumen, extracellular space, hemoglobin complex, mitochondrial matrix, and striated muscle myosin thick filament. MF analysis demonstrated that DE-mRNAs target genes were significantly enriched in aliphatic-amine oxidase activity, oxygen oxidoreductase activity, peptidase activity, oxygen transporter activity, heme binding, serine-type endopeptidase activity, and cytokine binding. Figure 3G, H illustrates up-regulated mRNAs enriched KEGG pathways: hsa04742: Taste transduction; hsa05410: Hypertrophic cardiomyopathy; hsa05414: Dilated cardiomyopathy; hsa00360: Phenylalanine metabolism. Down-regulations were enriched in hsa04613: Neutrophil extracellular trap formation; hsa05202: Transcriptional misregulation; hsa05150: Staphylococcus aureus infection; and hsa04621: NOD-like receptor signaling pathway. These results suggested a linkage between exercise and inflammation response and material and energy metabolism.

### Protein–protein interaction (PPI) network and module extraction

**Fig. 4:**
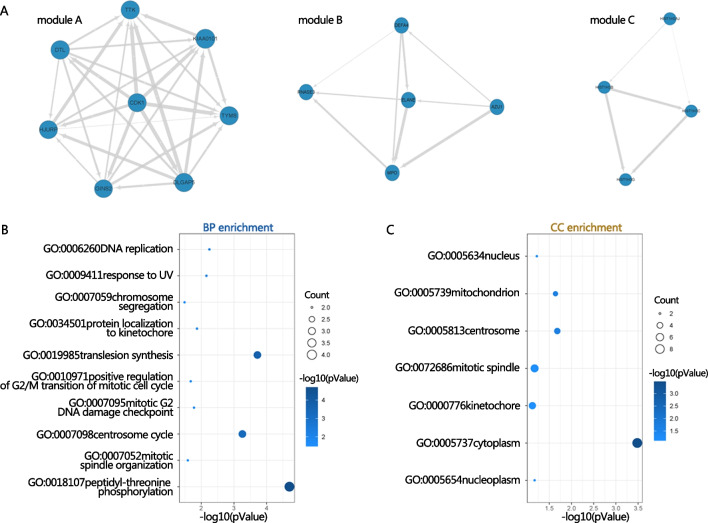
Three modules extracted from protein–protein interaction (PPI) network. A total of 3 modules were identified in the PPI network using the MCODE tool in Cytoscape software (**A**–**C**). Blue circles represent down regulated genes. Top 5 GO-BP (**D**) and CC (**E**) terms enriched by genes in those three modules

There are 43 nodes and 66 interaction pairs in the De-mRNA-based PPI network. We were successful in separating three sub-network modules from the PPI network using the MCODE Cytoscape plug-in. The major genes identified by nodes with a high topological score were all down-regulated (Fig. 4), including Cyclin-Dependent Kinase 1 (CDK1), TTK Protein Kinase (TTK), Holliday Junction Recognition Protein (HJURP), DLG Associated Protein 5 (DLGAP5), PCNA Clamp Associated Factor (KIAA0101, PCLAF), GINS Complex Subunit 2 (GINS2), Thymidylate synthase (TYMS), Denticleless E3 Ubiquitin Protein Ligase Homolog (DTL), Kinesin Family Member C1 (KIFC1), and Elastase Neutrophil Expressed (ELANE). Module I (score = 8) contained eight nodes and 28 edges, Module II (score = 4.5) contained five nodes and 28 edges, Module III (score = 3.3) contained four nodes and five edges. For the GO enrichment analysis, three modules of genes were used. The genes in Module I were significantly involved in GO:0007059 ~ chromosome segregation, GO:0034501 ~ protein localization to kinetochore, GO:0010971 ~ positive regulation of G2/M transition of mitotic cell cycle, and GO:0006260 ~ DNA replication. Genes from Module II were heavily engaged in GO: 0019731 ~ antibacterial humoral response, GO:0050829 ~ defense response to Gram-negative bacterium, and GO:0002227 ~ innate immune response in mucosa. Module III were involved in GO:0060968 ~ regulation of gene silencing, GO:0040029 ~ regulation of gene expression, GO:0032200 ~ telomere organization, GO:0006335 ~ DNA replication-dependent nucleosome assembly, and GO:0006334 ~ nucleosome assembly. Results of the top five terms of GO-BP in each module were selected for display in Fig. 4.

### Gene set variation analysis (GSVA) of expressed mRNAs

**Fig. 5:**
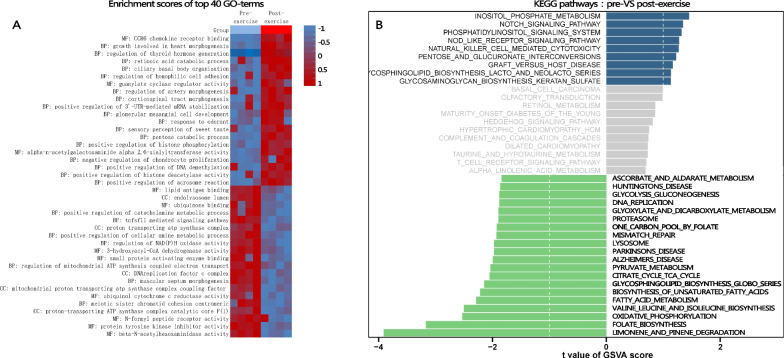
Gene function enrichment scores and pathway analysis. **A** the heatmap showed the enrichment scores of different gene sets of each COPD patient pre-and post-exercise. **B** Diverging bars showed enrichment analysis of KEGG pathways on these gene sets

GSVA was used to determine the enrichment score of each patient. Of 86 differently scored GO gene sets, 54 gene sets (94.56%) were down-regulated and 32 gene sets were up-regulated in the post-exercise group compared to the pre-exercise COPD group, at criteria of FC > 1.5, p-value < 0.05 (top 40 terms were shown in Fig. 5A). BP analysis indicated that these gene sets were enriched in growth involved in heart morphogenesis, material and energy metabolism, and cell adhesion. The CC analysis further verified that the gene sets were enriched in structural and functional components of material and energy metabolism, especially the mitochondrial-related energy metabolism. Moreover, the MF analysis indicated that the gene sets were enriched in CCR6 chemokine receptor biding, Guanylate cyclase regulator activity, alpha-N-acetylgalactosaminide alpha-2, 6-sialyltransferase activity, BMP receptor activity, and TGF-βreceptor activity. 570 genes were enriched using these terms, among which SCN1B, STPG4, DTL, TTK, FPR3, CDK1, HJURP, and GINS2 were also included in 86 De-mRNAs.

According to KEGG pathway enrichment analysis, we found 12 pathways were suppressed compared to pre-exercise COPD patients at criteria P < 0.05 (Fig. 5B). The most enriched terms in COPD following exercise were: limonene and pinene degradation, folate biosynthesis, oxidative phosphorylation, valine leucine and isoleucine biosynthesis, fatty acid metabolism, biosynthesis of unsaturated fatty acids, glycosphingolipid biosynthesis globo series, citrate cycle TCA cycle, pyruvate metabolism, and lysosome. 492 genes were enriched in these pathways, among which IGF2R, NDUFAB1, NDUFS8, PLCB4, and SCD were also included in DE-mRNAs of our study at criteria FC > 1.5. Of note, PLCB4 was DE-mRNA at criteria FC > 2. Aerobic exercise regulated metabolic pathways in COPD patients, particularly the energy metabolism pathways. In summary, our study offered a thorough explanation of the effect of exercise on the transcriptome in COPD patients, who were characterized by a lack of energy supply.

### Enrichment analysis of target genes associated with miRNA, lncRNA, and circRNA

**Fig. 6:**
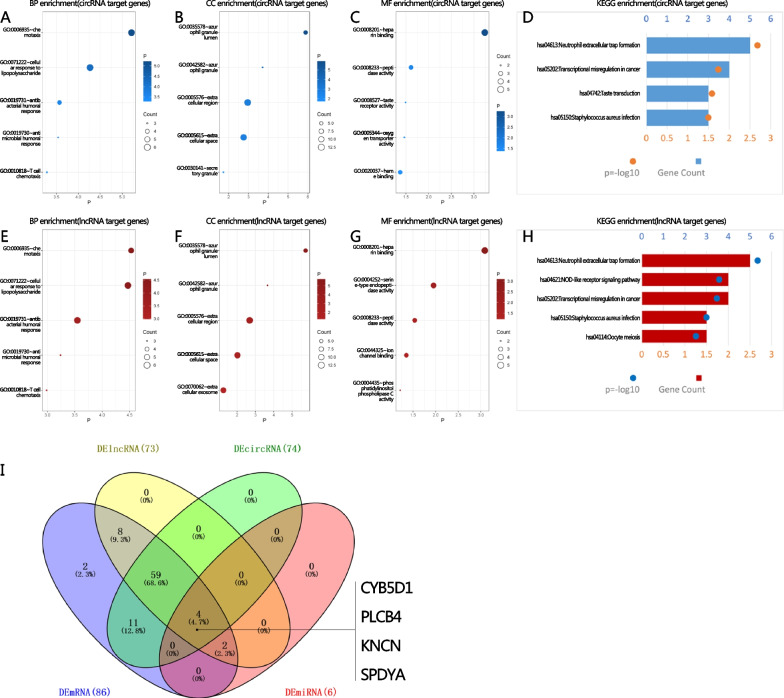
Enrichment analysis of target genes of DE-lncRNAs and DE-circRNAs. Host genes of DE-circRNA enriched in BP (**A**), CC (**B**), MF (**C**) terms and KEGG pathways (**D**); Target genes of DE-lncRNA enriched in BP (**E**), CC (**F**), MF (**G**) terms and KEGG pathways (**H**); intersection mRNAs (**I**) that were identified by Venn diagram using prediction platforms and DE-mRNAs in our study

A growing body of data suggests that complementary hybrids between lncRNAs and co-expressed mRNAs can interact with one another from both adjacent and distant places. CircRNAs also participate in a variety of biological processes through a number of methods, due to their distinct shapes and characteristics. Figure 6 depicts the intersections of DE-lncRNA-target mRNAs, DE-circRNA-host genes, and DEmiRNA-target mRNAs. Four trustworthy core mRNAs that were identified by Venn diagram: Cytochrome B5 Domain Containing 1 (CYB5D1), Kinocilin (KNCN), Phospholipase C Beta 4 (PLCB4), and Speedy/RINGO Cell Cycle Regulator Family Member A (SPDYA). Based on the mRNAs involved in the DE-miRNA-DE-mRNA regulatory relationship, DE-lncRNA-DE-mRNA and DE-circRNA-DEmRNA co-expression relationship, GO and KEGG enrichment analysis was performed again, and the results are displayed in a bubble diagram (Fig. 6I). The results showed that the genes most enriched in the inflammatory response following exercise training. Future research may use these co-expression networks to identify possible functional connections between ncRNAs and mRNAs.

### CeRNA network construction

**Fig. 7:**
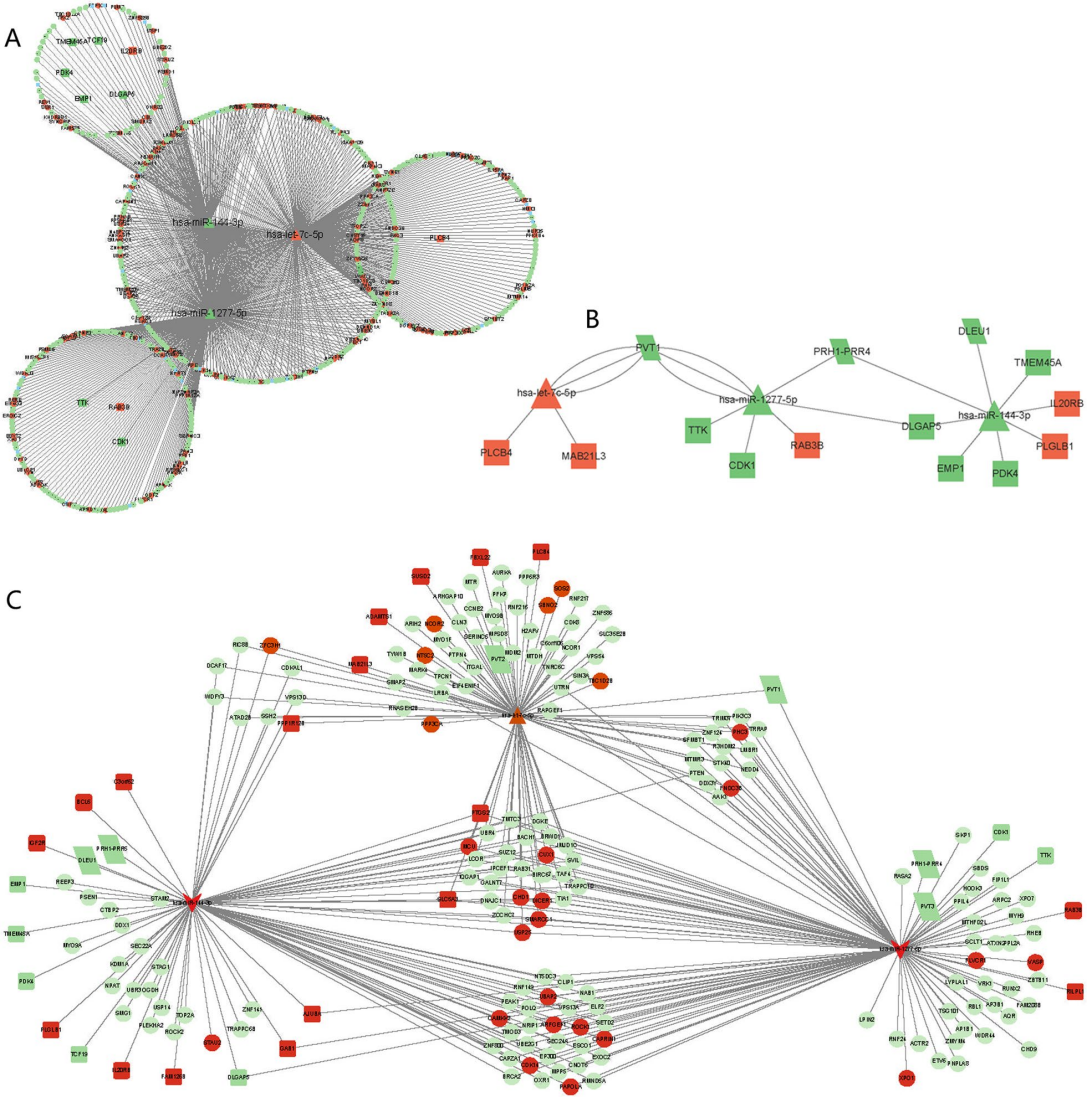
The competing expression networks. **A** shows the ceRNA network of lncRNA-miRNA-mRNA, **B** shows circRNA-miRNA-mRNA ceRNA network, **C** shows the circRNA-lncRNA-miRNA-mRNA ceRNA network. Orange-red color represents upregulated genes, green color indicates downregulated genes. Circle shape indicates circRNAs, quadrilateral indicates lncRNAs, triangle indicates miRNAs

By combining expression profiling of differentially expressed circRNAs, miRNAs, and mRNAs, a ceRNA network was built to thoroughly study the impact of ceRNA regulation on gene levels in COPD following exercise training. As shown in Fig. 7A, there are 1238 interaction relationships of circRNA-miRNA-mRNA: 161 up-circRNAs and 336 downcircRNAs;

18 upregulated mRNA and seven downregulated mRNAs; and one upregulated miRNA and two downregulated miRNAs. Figure 7B showed 31 lncRNA-miRNA-mRNA interactions, including one upregulated miRNA and two downregulated miRNAs, 18 upregulated mRNAs, and seven downregulated lncRNAs. Further, we integrated these two networks and concentrated on screening circRNAs, lncRNAs, and mRNAs that were regulated by the same miRNAs.Finally, 312 interaction pairs were discovered (Fig. 7C), including 176 circRNAs (26 upregulated and 150 downregulated), 25 mRNAs (18 upregulated and seven downregulated), three miRNAs (hsa-miR-1277-5p and hsa-miR-144-3p, downregulated; hsa-let-7c-5p, upregulated), and six downregulated lncRNAs,

### Verification of the expression changes of key genes

According to our results, DE-mRNAs were linked to mitochondrial metabolism and inflammatory responses, both of which are compromised in COPD. Additional research on the crucial molecules involved in PR could provide more insight into the mechanisms underlying the metabolic remodeling of COPD prompted by aerobic exercise. Therefore, we used GEO statabase to validate the differently expression genes, where the screening threshold was set at p < 0.05. A total of 8545 different expression mRNAs were obtained in GSE76705, which comprised 144 COPD samples and 85 controls. Finally, in contrast to the DE-mRNA produced by our analysis, 30 DE-mRNAs were found (see Venn map [41] in Figure S1), including genes with greater levels of enrichment in the PPI modules and higher GSVA scores: ELANE, SCN1B. Additionally, we noticed that two down-regulated and 17 up-regulated (such as: ELANE, DEFA4, MPO, CPA3, AZU1, CCL23 in GSE76075) genes’ expression levels reversed in COPD patients after aerobic activity in our study. However, aerobic exercise did not reverse levels of one down-regulated and 10 up-regulated genes in COPD patients. In fact, they were even higher or lower (such as: SCN1B, TIAF1). Notably, DTL, CDK1 and PLCB4 were not changed in GSE 146560 COPD samples when compared to normal controls, but the expression levels changed after exercise. However, these three genes were not detected in GSE76075; further studies are needed to address this.

**Fig. 8:**
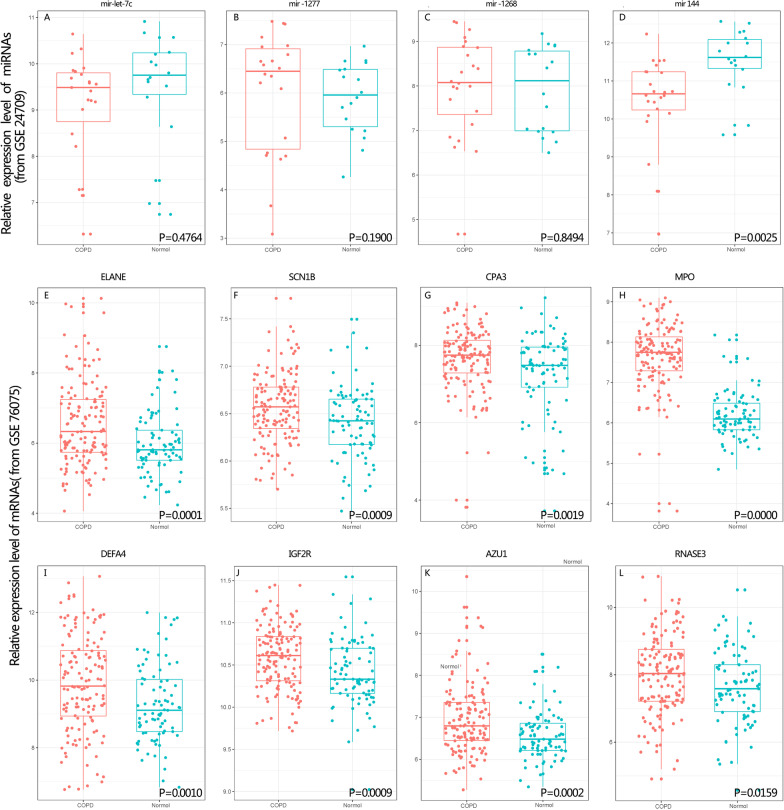
GEO database verification of mRNAs and miRNAs. **A**–**D**: The miRNA expression levels that are different between COPD and normal controls. **E**–**L** In GES76075, genes were found to be significantly increased in COPD patients versus normal controls. The significance of the difference was evaluated with an unpaired t test

GSE24709 and GSE 61741 datasets were used for mirRNAs data validation, 4/8 (50%) DE-miRNAs in our study were expressed, including hsa-miR-144, has-miR-1268, hsa-let-7c-5p, and hsa-miR-1277(the levels of these genes in GSE 24709 were shown in Fig. 8). However, only hsa-miR-144 was differentially expressed in these two GEO data compared to normal controls. The difference in results may be due to sample or threshold differences. However it worth to be researched in the future study.

**Fig. 9:**
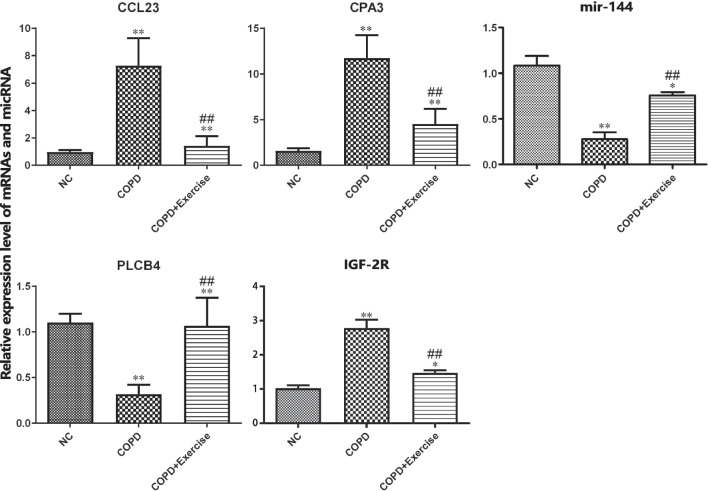
Verification of Gene Expressions via RT-PCR. **A**–**E** RT-qPCR detection show the expression of CCL23, CPA3, PLCB4, IGF2R and miR-144. Data are means ± SD. **P < 0.01, ***P < 0.001 vs. control. ^##^P < 0.01, ^###^P < 0.001 vs. COPD

High potential therapeutic targets that were DE-RNAs representatives were chosen for RT-PCR investigation in order to confirm the results from RNA-seq. As shown in Fig. 9, 5 major regulatory DE-RNAs were selected for qRT-PCR verification, including PLCB4, CCL23, CPA3, Mir-144, and its target gene IGF2R. The housekeeping gene GAPDH was used as the endogenous control. Patient characteristics are shown in Additional file 1: Table S1. These DE-RNAs exhibited the same tendency between the RNA-seq analysis and qRT-PCR results, which suggested that our transcriptome analysis was accurate and dependable.

## Conclusion

COPD is a heterogeneous disease, although pharmacological therapies for COPD have improved, they produce insufficient results. Exercise training is a vital tool in the fight against the global epidemic of aging and metabolic disease^3^, including COPD. Numerous studies have shown the effects of exercise on the immune system. Regular exercise performed at moderate intensity leads to an anti-inflammatory, anti-infection, and controlled immunological metabolic state [42, 43]. However, the exact mechanism that underlies its effects on COPD remains unclear. Since the basis of exercise limitation in COPD patients is breathlessness [16], we used personalized, precise exercise training to inhibit these respiratory symptoms to achieve the daily exercise volume. Our study found individualized aerobic exercise training improved peak VO2, CAT score, and 6MWT (see Fig. 1), compared to healthy cohorts in a meta-analysis study [44]. According to the findings, personalized, precise exercise training is appropriate for COPD and has promising future prospects. But the mechanism by which exercise increases exercise tolerance and improves respiratory symptoms remains unknown.

We employed a whole-transcriptome sequencing technique to investigate the potential role of RNAs in COPD following exercise training and provided a comprehensive look at COPD patients' pre- and post-exercise caused RNA-level modifications. 570 lncRNAs, 2064 circRNAs, eight miRNAs, and 86 mRNAs were found to have significantly altered expression in COPD patients pre- and post-exercise using log_2_FC ≥ 1 and p-value < 0.05 as the criteria (Fig. 2). Direct function enrichment analysis revealed that DE-genes were involved in brown fat cell differentiation, muscle contraction, G-protein coupled receptor signaling pathway, and detection of chemical stimulus, T cell chemotaxis, DNA replication, and antibacterial and antimicrobial humoral response. PPI network analysis of these DE-genes identified several hub gene: CDK1, TTK, HJURP, DLGAP5, PLCAF, GINS2, TYMS, DTL, KIFC1, and ELANE. Enrichment analysis of those genes related to the biologic process of DNA replication, chromosome segregation, telomere organization, and anti- inflammation. We also performed GSVA analysis with non-zero expressed mRNAs in each patient. Eleven hub genes (DTL, TTK, FPR3, CDK1, HJURP, GINS2, IGF2R, NDUFAB1, NDUFS8, PLCB4, and SCD) and some vital pathways linked to regular exercise training were discovered: chemokine receptor biding, tricarboxylic acid (TCA) cycle, fatty acid metabolism, and oxidative phosphorylation, which were known to be associated with the pathophysiology of COPD [3, 45, 45]. In addition, enrichment analysis of co-expression genes linked to miRNA, lncRNA, and circRNA also identified the function of anti-infection and T-cell chemotaxis. According to the ceRNA complex network, we found hsa-miR-144-3p, hsa-miR-1277-5p, and hsa-miR-7c-5p were significantly enriched in. Moreover, majority of the DE-mRNAs and a small number of miRNAs were effectively confirmed using GEO data. Different threshold choices or sample variances may be the cause of the discrepancy between our sequencing results and GEO data.

Despite limited evidence, the key genes listed in DE-mRNAs from our results may be linked to RNA replication immune defense, anti-inflammation, mitochondrial functions, and ATP and protein degradation processes. Genes such as CDK1, TTK, HJURP, GINS2, DTL, ELANE, FPR3, PLCB4, and IGF2R may contribute to the pathology of of COPD. CDK1, a member of the cyclin-dependent kinase family which is up-regulated in several cancers by regulating cell cycle progression and activating of JAK/STAT3 signaling[46, 47], participated in the pathogenesis of pulmonary arterial hypertension (PAH) by influencing mitochondrial dynamics and the cell cycle [48]. Further studies are needed to validate them as a target for cancer therapy. TTK is a critical component of the spindle assembly checkpoint [49]. It is a biomarker for non-small cell lung cancer prognosis, and its overexpression accelerates the tumor’s progression [50, 51]. HJURP, a centromeric protein (chaperone), has been shown to increase in lung tumors and COPD and is essential for the insertion and maintenance of the histone H3-like variation CENPA at centromeres [52, 53]. GINS2 promoted cell proliferation, migration, invasion, and EMT via modulating PI3K/Akt and MEK/ERK signaling pathways [54], GINS2 knock-down stimulated inflammation and apoptosis in microglia [55]. DTL, a homolog of E3 ubiquitin ligase that belongs to the DCAF protein family, was reported to enhance the motility, proliferation, and invasion of cancer cells [56, 57], and also significantly decrease total glucose consumption and lactate production [58]. ELANE, a factor that contributes to a protease-antiprotease imbalance and may cause inflammatory lung illnesses [59], induces autophagy, which in turn induces lung epithelial cell apoptosis and pulmonary emphysema through the overexpression of PGF [60]. Further research is needed to determine if RNAs with differential expression are involved in these biological activities.

RNA-seq-based networks have proven to be a valuable tool for investigating functional noncoding RNAs and their functional mechanisms in many disease models. We discovered that has-miR-144-3p plays an important role in the ceRNA network. Evidence shows that miR-144-3p, which is improperly regulated, suppresses tumor growth in a variety of cancer types [61]. In addition, miR-144-3p was downregulated in the peripheral blood of COPD patients compared to normal controls according to GSE24709 and GSE6141. However, our results indicated that miR-144-3p was down-regulated post-exercise when compared to pre-exercise COPD. Anti-mir-144-loaded extracellular vesicles was proven to protect against obstructive sleep apnea or chronic intermittent hypoxia-associated endothelial dysfunction [62].The miR-144 family was reported to target NF-kB pathways and play a pro-inflammatory role in coronary artery disease [63]. Thus, we speculated that exercise training may improve progression of COPD by regulating the expression of miR-144-3p.

Interestingly, up-regulated hsa-let-7c and down-regulated hsa-miR-1277 post-exercise in COPD, which were validated as expressed but without difference compared to normal controls in GEO data, play an important role in the ceRNA network of our study. Evidence suggests that overexpression hsa-miR-1277 could ameliorate IL-1β-induced CHON-001 cell injury and inhibit the progression of Parkinson’s disease [64, 65], but these studies were all in vitro. Future mechanistic investigations are thus necessary to ascertain the impact of exercise on COPD and the function of miR-144-3p and other ncRNAs. However, because of the paucity of study in this field, a significant portion of the DE-lncRNAs and DE-circRNAs were not previously identified. In general, the discovery of RNAs changes following exercise improves our comprehension of the cardiopulmonary-regulation mechanisms of aerobic exercise in COPD, which may enhance the effectiveness of this non-pharmacological intervention and result in the discovery of novel alternative therapeutic targets for COPD patients.

In summary, while the influence of exercise on multiple organs is well documented, our knowledge of how this occurs at the cellular and molecular level is mostly limited to skeletal muscle. According to GO enrichment, KEGG, and GSVA analysis, this comprehensive study of noncoding RNAs and mRNAs uncovers regulatory pathways and key DE-genes involved in the effectiveness of aerobic exercise on COPD. Furthermore, co-expression networks (lncRNA–miRNA–mRNA and circRNA–miRNA–mRNA) were constructed to understand the regulatory roles of these mRNAs and ncRNAs. The observed DE-mRNAs and DE-ncRNAs may provide the foundation for understanding the genetic basis and ceRNA mechanism of exercise in COPD. The results provide molecular insights related to the effects of exercise on COPD and inform future therapeutic selection. Further research into the molecular mechanisms underlying the expression changes on differentially expressed mRNAs and ncRNAs may reveal more RNA therapeutic targets.

The results of this study offer implications for further investigation. First, more COPD patients who volunteer for 12 weeks of supervised exercise are expected to participate in RNA sequencing and bioinformatics analysis based on sample size estimation. This will enhance research methodology for highly confident differential expression identification. Second, future comprehensive investigations involving in vivo and in vitro trials are necessary because the RNA regulatory networks and data validation were solely based on bioinformatics predictions and GEO database, and lacking sufficient sample sizes for verification. Research on the potential functions and evolutionary conservation of RNAs can benefit from using COPD model exercises. Moreover, repeatability in different COPD phenotypes and RNA alterations in the current study are incomplete. Our team is researching these key RNAs’ repeatability and mechanisms post-exercise in COPD models, as well as expanding the COPD participant pool.

## Supplementary Information

**Additional file 1: Table S1**. Information of patients for RT-qPCR validation.

## Data Availability

The datasets are available in Chinese Clinical Trial Registry, the accession number is ChiCTR2100053232. The datasets also are available in GEO database.

## Abbreviations

COPD: Chronic obstructive pulmonary disease
lncRNAs: Long non-coding RNAs
DE: Diferent expression
circRNA: Circular RNA
miRNA: MicroRNA
mRNA: Message RNA
ncRNA: Non-coding RNA
ceRNA: Competing endogenous RNAs
FEV1: Forced expiratory volume in 1 s
FVC: Forced vital capacity
GSVA: Gene set variation analysis
