# Supplementary material for: Regulation of whole-transcriptome sequencing expression in COPD after personalized precise exercise training: a pilot study
Source: Respir Res. 2023 Jun 13;24:156. doi: 10.1186/s12931-023-02461-y (PMC10262399; doi:10.1186/s12931-023-02461-y)
Supplement: Supplementary file 1 — Additional file 1: Table S1. Information of patients for RT-qPCR validation. [file 12931_2023_2461_MOESM1_ESM.pdf]

Table S1: Information of patients for RT-qPCR validation

	Normal control (n=10)	COPD (n=10)	COPD+Exercise (n=10)	P-value
Demographic data				
Age, years	59.90±2.51	62.30±24.03	/	0.127
BMI, Kg/m^2	26.31±2.83	24.04±4.92	/	0.222
Male gender	9(90.0)	9(90.0)	/	>0.9
Smoking History				
Current smoker	2(20.00)	1(10.00)	/	0.531
Ex-smoker	5(50.00)	8(80.00)	/	0.159
Smoking, (pack-years)	37.10±13.77	34.30±12.78	/	0.642
Lung Function and Cardiopulmonary Exercise Test				
FEV1,%pred	91.80±5.75	42.59±13.41**	41.88±14.85	0.000
FEV1/FVC, %	87.20±4.78	41.47±19.87**	40.61±19.67	0.000
Peak-VO2	1350.50±87.26	1001.80±370.14**	1121.90±369.31** ##	0.050
CAT score				
	/	25.20±6.58	15.50±6.17 ##	
6MWD				
	402.60±9.01	285.50±88.79**	326.90±76.17** ##	0.002

Data are presented as mean (SD) or number (percentage); The difference between the Three groups was analyzed by One-way ANOVA; BMI, body mass index; FEV1: forced expiratory volume during the first second; FVC: forced vital capacity, VO2: oxygen consumption, HR: heart rate, SPO2:oxygen saturation, AT: anaerobic threshold; CAT: COPD assessment test; 6MWD:6 minutes walking distance. (** P < 0.001, compared with control group; ## P < 0.001 compared with COPD group
